# A life-course approach to health: synergy with sustainable development goals

**DOI:** 10.2471/BLT.17.198358

**Published:** 2017-11-23

**Authors:** Shyama Kuruvilla, Ritu Sadana, Eugenio Villar Montesinos, John Beard, Jennifer Franz Vasdeki, Islene Araujo de Carvalho, Rebekah Bosco Thomas, Marie-Noel Brunne Drisse, Bernadette Daelmans, Tracey Goodman, Theadora Koller, Alana Officer, Joanna Vogel, Nicole Valentine, Emily Wootton, Anshu Banerjee, Veronica Magar, Maria Neira, Jean Marie Okwo Bele, Anne Marie Worning, Flavia Bustreo

**Affiliations:** a Office of the Assistant Director-General, Family, Women’s and Children’s Health, World Health Organization, avenue Appia 20, 1211 Geneva 27, Switzerland.; b Department of Ageing and Life Course, World Health Organization, Geneva, Switzerland.; c Department of Public Health, Environmental and Social Determinants of Health, World Health Organization, Geneva, Switzerland.; d Department of Gender, Equity and Human Rights, World Health Organization, Geneva, Switzerland.; e Department of Maternal, Newborn, Child and Adolescent Health, World Health Organization, Geneva, Switzerland.; f Department of Immunization, Vaccines and Biologicals, World Health Organization, Geneva, Switzerland.; g Office of the Director-General, World Health Organization, Geneva, Switzerland.

## Abstract

A life-course approach to health encompasses strategies across individuals’ lives that optimize their functional ability (taking into account the interdependence of individual, social, environmental, temporal and intergenerational factors), thereby enabling well-being and the realization of rights. The approach is a perfect fit with efforts to achieve universal health coverage and meet the sustainable development goals (SDGs). Properly applied, a life-course approach can increase the effectiveness of the former and help realize the vision of the latter, especially in ensuring health and well-being for all at all ages. Its implementation requires a shared understanding by individuals and societies of how health is shaped by multiple factors throughout life and across generations. Most studies have focused on noncommunicable disease and ageing populations in high-income countries and on epidemiological, theoretical and clinical issues. The aim of this article is to show how the life-course approach to health can be extended to all age groups, health topics and countries by building on a synthesis of existing scientific evidence, experience in different countries and advances in health strategies and programmes. A conceptual framework for the approach is presented along with implications for implementation in the areas of: (i) policy and investment; (ii) health services and systems; (iii) local, multisectoral and multistakeholder action; and (iv) measurement, monitoring and research. The SDGs provide a unique context for applying a holistic, multisectoral approach to achieving transformative outcomes for people, prosperity and the environment. A life-course approach can reinforce these efforts, particularly given its emphasis on rights and equity.

## Résumé

Une approche sanitaire fondée sur le parcours de vie englobe des stratégies tout au long de la vie des individus qui optimisent leur capacité fonctionnelle (en prenant en compte l'interdépendance de facteurs individuels, sociaux, environnementaux, temporels et intergénérationnels), assurant ainsi le bien-être et l'exercice des droits. Cette approche s'inscrit parfaitement dans les efforts déployés pour parvenir à une couverture sanitaire universelle et atteindre les objectifs de développement durable (ODD). Lorsqu'elle est correctement appliquée, une approche fondée sur le parcours de vie peut accroître l'efficacité de la première et aider à concrétiser l'ambition des seconds, en assurant notamment la santé et le bien-être pour tous à tous les âges. Sa mise en œuvre exige une compréhension commune par les individus et les sociétés de la manière dont la santé est façonnée par de multiples facteurs tout au long de la vie et d'une génération à l'autre. La plupart des études réalisées ont porté sur des maladies non transmissibles et le vieillissement des populations dans les pays à revenu élevé, ainsi que sur des aspects épidémiologiques, théoriques et cliniques. L'objectif de cet article est de montrer que l'approche sanitaire fondée sur le parcours de vie peut être élargie à toutes les tranches d'âge, toutes les questions de santé et tous les pays en s'appuyant sur une synthèse des données scientifiques existantes, les expériences de différents pays et l'avancement des stratégies et programmes en matière de santé. Un cadre conceptuel de l'approche est présenté ainsi que les conséquences de sa mise en œuvre sur: (i) la politique et l'investissement; (ii) les services et systèmes de santé; (iii) les actions locales, multisectorielles et multipartites; et (iv) les mesures, la surveillance et la recherche. Les ODD fournissent un contexte unique pour l'application d'une approche globale et multisectorielle en vue d'obtenir des résultats porteurs de transformation pour les individus, la prospérité et l'environnement. Une approche fondée sur le parcours de vie peut renforcer ces efforts, notamment parce qu'elle met l'accent sur les droits et l'équité.

## Resumen

Un enfoque basado en la salud para toda la vida engloba estrategias durante la vida de las personas, que optimizan su capacidad funcional (teniendo en cuenta la interdependencia de los factores individuales, sociales, ambientales, temporales e intergeneracionales), permitiendo así el bienestar y la realización de los derechos. El enfoque encaja perfectamente con los esfuerzos por lograr una cobertura sanitaria universal y cumplir los objetivos de desarrollo sostenible (ODS). Si se aplica correctamente, un enfoque para toda la vida puede aumentar la eficacia del primero y ayudar a alcanzar la visión de este último, especialmente para garantizar la salud y el bienestar en todas las edades. Su aplicación requiere una comprensión compartida entre individuos y sociedades sobre cómo la salud depende de múltiples factores presentes a lo largo de la vida y entre generaciones. La mayoría de los estudios se han centrado en las enfermedades no contagiosas, en el envejecimiento de la población en los países con ingresos altos y en cuestiones epidemiológicas, teóricas y clínicas. El objetivo de este artículo es mostrar cómo el enfoque basado en la salud para toda la vida se puede extender a todos los grupos de edades, temas de salud y países, mediante la creación de una síntesis de las pruebas científicas existentes, la experiencia en diferentes países y los avances en estrategias y programas de salud. Se presenta un marco conceptual del enfoque junto con las implicaciones para la aplicación en los siguientes campos: (i) política e inversión; (ii) servicios y sistemas de salud; (iii) acción local, multisectorial y de varias partes interesadas; y (iv) medición, supervisión e investigación. Los ODS proporcionan un contexto único para aplicar un enfoque holístico y multisectorial a fin de alcanzar unos resultados transformadores para las personas, la prosperidad y el medio ambiente. Un enfoque para toda la vida puede intensificar estos esfuerzos, sobre todo por su énfasis en los derechos y la equidad.

## ملخص

يتضمن نهج مسار الحياة للتمتع بالصحة مجموعة من الاستراتيجيات على مدار حياة الأفراد وهي تعمل على تحسين قدرتهم الوظيفية (مع مراعاة الترابط بين العوامل الفردية، والاجتماعية، والبيئية، والزمانية، وبين الأجيال المختلفة)، وهو الأمر الذي يتيح التمتع بالصحة والعافية وإعمال الحقوق. ويعد هذا النهج مناسبًا تمامًا للجهود الرامية إلى تحقيق تغطية صحية شاملة وتلبية أهداف التنمية المستدامة (SDG). يمكن أن ينتج عن تطبيق نهج مسار الحياة بشكلٍ صحيح، زيادة فعالية الخدمة الأولى والمساعدة على تحقيق رؤية الأهداف الأخيرة المذكورة، وخصوصًا في ضمان التمتع بالصحة والعافية لجميع الأشخاص بكافة الأعمار. ويتطلب تنفيذه وجود تفاهم مشترك بين الأفراد والمجتمعات حول كيفية تكوين مبادئ الصحة من خلال العديد من العوامل على مدار الحياة وعبر مختلف الأجيال. لقد ركزت معظم الدراسات على الأمراض غير المعدية، وعلى السكان المسنين في البلدان مرتفعة الدخل، وعلى المشكلات الوبائية والنظرية والسريرية. إن الهدف من هذه المقالة هو توضيح كيف يمكن تمديد نطاق نهج مسار الحياة للتمتع بالصحة ليشمل جميع الفئات العمرية، والموضوعات الصحية، والبلدان من خلال البناء على توليفة من الأدلة العلمية المثبتة بالفعل، والخبرة في مختلف البلدان، وأوجه التقدم المحرز في الاستراتيجيات والبرامج الصحية. يتم تقديم إطارًا مفاهيميًا لهذا النهج إلى جانب ذكر الآثار المترتبة على تنفيذه في المجالات التالية: (1) السياسة والاستثمار؛ (2) والخدمات والنظم الصحية؛ (3) والإجراءات التي يتم اتخاذها على المستوى المحلي والقطاعات المتعددة والجهات المعنية المتعددة؛ (4) والقياس والرصد والبحث. توفر أهداف التنمية المستدامة سياقًا فريدًا من نوعه لتطبيق نهج شامل عبر العديد من القطاعات لتحقيق النتائج التحويلية للأشخاص والرخاء والبيئة. ويمكن لنهج مسار الحياة أن يعزز هذه الجهود، لا سيما بالنظر إلى تركيزه على الحقوق والمساواة.

## 摘要

种健康的生活方式，包括优化机能水平的个人生活策略（考虑到个人、社会、环境、时间和代际因素之间的相互依赖性），从而实现福祉和权利。该方式完全符合实现全民健康保障和可持续发展目标。适当采用这种生活方式可以提高前者的有效性，并有助于实现后者的愿景，特别是在确保所有年龄段人民的健康和幸福方面。它的实施需要个人和社会共同理解：在整个生命和几代人中，健康是如何受到多种因素影响的。大多数研究都集中在高收入国家的非传染性疾病和老龄化人群以及流行病学、理论和临床问题上。本文旨在展示如何通过综合现有的科学实证、不同国家的经验以及卫生策略和方案的进展，将该健康的生活方式推广到所有年龄段、健康主题和国家。展示该方式的概念框架以及实施对下列领域产生的影响：(i) 政策和投资；(ii) 卫生服务和系统；(iii) 地方、多部门和多方利益相关者的行动；(iv) 测量、监测和研究。可持续发展目标为采用全面、多部门的方法实现人民、繁荣和环境的变革成果提供了独特的环境。一种生活方式可以加强这些努力，特别是考虑到对权利和公平的重视。

## Резюме

Подход к здоровью, рассчитанный на всю продолжительность жизни человека, включает в себя применяемые на протяжении всей жизни людей стратегии, которые оптимизируют их функциональные способности (с учетом взаимозависимости индивидуальных, социальных, экологических, временных и межпоколенческих факторов), тем самым обеспечивая благополучие и реализацию прав. Этот подход идеально сочетается с усилиями по достижению всеобщего охвата медико-санитарным обслуживанием и целей в области устойчивого развития (ЦУР). Правильно применяемый подход на протяжении всей жизни может повысить эффективность первого и помочь реализовать концепцию последних, особенно в обеспечении здоровья и благополучия для всех людей любого возраста. Его реализация требует общего понимания отдельными людьми и обществом того, как под воздействием нескольких факторов формируется здоровье в течение всей жизни и из поколения в поколение. В большинстве исследований основное внимание уделялось неинфекционным болезням и пожилой группе населения в странах с высоким уровнем доходов, а также эпидемиологическим, теоретическим и клиническим вопросам. Цель этой статьи — показать, как подход к здоровью, рассчитанный на всю продолжительность жизни, может быть распространен на все возрастные группы, вопросы относительно здравоохранения и страны, опираясь на обобщение существующих научных данных, опыта в разных странах и достижений в стратегиях и программах здравоохранения. Представлены концептуальные схемы подхода, а также последствия для реализации в следующих областях: (i) политика и инвестиции; (ii) медико-санитарное обслуживание и система здравоохранения; (iii) местная, многосекторальная деятельность с участием многих заинтересованных сторон и (iv) измерения, мониторинг и исследования. ЦУР представляют собой уникальный фон для применения целостного многосекторального подхода к достижению таких результатов, которые оказывают преобразующее воздействие на людей, их благосостояние и окружающую среду. Данный подход может подкреплять эти усилия, особенно с учетом его акцента на правах и справедливости.

## Introduction

The right to the highest attainable standard of health for all people, is enshrined in the World Health Organization’s (WHO) constitution and in the United Nations’ (UN’s) human rights framework.^1^^,^^2^ Subsequent international declarations emphasize that health is interlinked with peace, development and the environment.^1^^,^^3^^–^^6^ The UN’s Sustainable Development Goal 3 (SDG 3) for 2030 is to “ensure healthy lives and promote well-being for all at all ages”.^7^ Adoption of the SDGs provides a unique opportunity to apply a holistic, people-centred, multisectoral approach to health and development, which is well aligned with a life-course approach to health.^8^^–^^12^ Properly applied, a life-course approach can help realize the vision of SDG 3, ensure universal health coverage (UHC) and achieve health and well-being for all at all ages.

To take advantage of this unique opportunity, we commissioned two reports on the evidence supporting a life course approach to health to inform policy and practice in the SDG era. The first focused on conceptual and operational implications and the second on epidemiology and the risk of chronic disease.^13^^,^^14^ These evidence syntheses showed that there remain barriers to implementing a life-course approach. There needs to be a better, shared understanding of how individuals’ health and well-being are shaped by multiple factors and how the risk of ill health can accumulate across life stages and generations.^8^^–^^10^^,^^13^^–^^16^ One obstacle is the current focus on single diseases or specific age groups, rather than holistically addressing health throughout life. Operational constraints include frequent shifts in political priorities, short-term policy and funding cycles, poor coordination between the actions of health care and other sectors of society in addressing social and environmental change (which may threaten both health and sustainable development) and limited measurement of health and well-being across individuals’ lives and generations.^12^^–^^14^^,^^17^ In addition, the evidence syntheses found that most studies of the life-course approach focused on noncommunicable diseases and healthy ageing in high-income countries – few considered other health topics, younger age groups or low- and middle-income countries.^13^ Further, the predominant themes were theoretical, epidemiological, research-based and clinical, with limited application to policy, planning and programme implementation.^13^^,^^14^

The aim of this article is to show how a life-course approach can be extended to all health topics, age groups and countries by building on a synthesis of existing scientific evidence, experience in different countries and advances in health strategies and programmes. Aligned with the SDGs and UHC, a life-course approach can facilitate the integration of individual, social, economic and environmental considerations.^7^ As recommended by the Lancet Commission on Planetary Health, operationalizing a life-course approach will involve “integrating the aim of sustained improvements in human health and well-being with the preservation of key natural systems, supported by good governance and appropriate policies”.^17^

First, we provide a conceptual framework for a life-course approach by presenting definitions of the key concepts required to build shared understanding – a prerequisite for collective action.^9^^,^^10^^,^^13^ This conceptual framework was developed by analysing and thematically categorizing the findings of the two evidence synthesis reports, which reviewed more than 200 publications, using a qualitative, narrative synthesis method.^13^^,^^14^^,^^18^ The conceptual framework was based on WHO’s World Report on Ageing and Health.^15^ Its approach to healthy ageing was extended to other age groups and health topics through consultations across WHO departments that took into account scientific and conceptual advances and updates in global strategies and programmes.^8^^–^^10^^,^^16^^,^^19^ Our synthesis method followed a multigrounded theory approach and included deductive and inductive analyses and triangulation of the different methods.^20^ Particular attention was paid to the implications of implementing a life-course approach to help achieve UHC and realize the SDGs.

## Conceptual framework

**Fig. 1:**
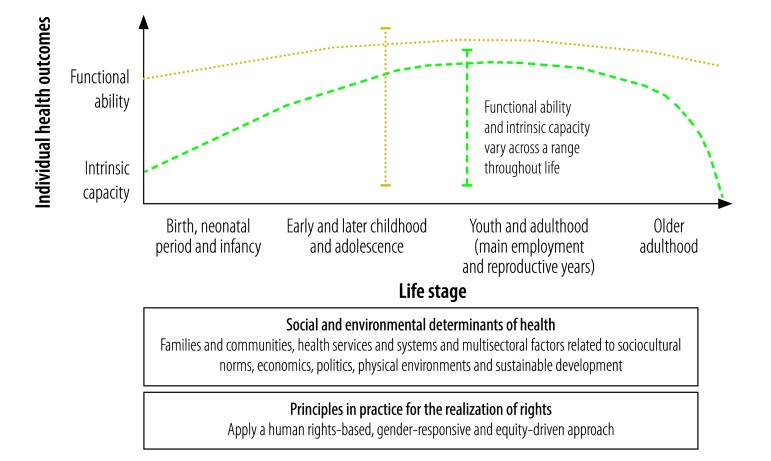
Conceptual framework for a life-course approach to health

As we defined it, life course approach to health optimizes the functional ability of individuals throughout life, enables well-being and realization of rights, and recognizes the critical interdependence of individual, intergenerational, social, environmental and temporal factors. Fig. 1 depicts the conceptual framework we developed. It is important to provide clear definitions of key concepts, such as: (i) functional ability; (ii) intrinsic capacity; (iii) well-being; (iv) the realization of rights; (v) life stage; (vi) resilience; (vii) risk; and (viii) the social and environmental determinants of health.

The main outcome of the life-course approach to health is functional ability, which is the sum of the individual and environmental attributes that enable a person to be or do what they have reason to value.^10^ Functional ability enables well-being at all ages and is interdependent with the realization of rights. For a neonate or infant, functional ability could be manifested by feeding well and playing; for older adults, by the ability to function independently without dependence on care. Functional ability is determined by the individual’s intrinsic capacity and physical and social environments and by the interaction between the individual and these environments.^10^ Intrinsic capacity is the sum of all physical and mental (including psychological) capacities.^10^ The concept of functional ability is consistent with the International Classification of Functioning, which defines functioning as a composite term for body functions and structures and the individual’s activities and participation.^21^ Additionally, functional ability takes into account the interaction between, and the interdependence of, individual, social and environmental determinants of health and the individual agency and collective actions required to ensure health and well-being throughout life. In Fig. 1, functional ability and intrinsic capacity are depicted as idealized arcs across the life course. Intrinsic capacity follows a biologically determined trajectory of physical and mental capacities. In contrast, functional ability can be optimized throughout life by a supportive environment. The vertical bars in Fig. 1 indicate that functional ability and intrinsic capacity can vary across a range at all life stages. This variability depends on the individual’s circumstances and on the critical events that influence health trajectories.

Well-being is a subjective state that can be evaluated across three domains: (i) perceived life satisfaction; (ii) emotions experienced; and (iii) self-realization and a sense of purpose or meaning.^22^ A meta-analysis of longitudinal studies indicated that subjective well-being is associated with lower mortality.^22^

Good health depends on, and enables, the realization of rights.^2^^,^^23^ Desired health outcomes should be achieved by applying human rights-based, gender-responsive and equity-driven approaches to policy-making and programme implementation. In addition to fulfilling legal obligations, this will help ensure better and more equitable health outcomes.^24^^,^^25^

On life stages, there are differing perspectives and a need for clarity. For example, questions exist about whether life begins at birth or earlier, about the most appropriate age ranges for different stages and about the specific events that influence health trajectories throughout life. Life stage definitions are influenced not only by chronological age, but also by sociocultural norms and the individual’s functional status.^13^^,^^14^ For practical reasons and for programming purposes, here the life course is regarded as starting at birth. Our timeline in Fig. 1 covers four broad stages: (i) birth, the neonatal period and infancy; (ii) early and later childhood and adolescence; (iii) youth and adulthood; and (iv) older adulthood. These four stages are based on programming approaches used in global health strategies.^8^^–^^10^^,^^16^^,^^19^ The preconception period and pregnancy, which occur during the reproductive years, are also important times during the life course. They can affect the next generation, as reflected in intrinsic capacity at birth.

Resilience to ill health and the risk of ill health accumulate throughout life and across generations.^13^^,^^14^ For example, 70% of preventable deaths from noncommunicable diseases in adults have been linked to risks encountered, and behaviours that started, during adolescence.^26^ In particular, 50% of mental health problems are established by the age of 14 years and 75%, by the age of 24 years.^27^ In addition to the critical events that shape an individual’s health trajectory, the number and sequence of exposures to risk and periods of increased susceptibility – some of which occur before birth or are genetically inherited – are also crucial.^12^^–^^14^^,^^16^

Social and environmental determinants of health include health systems, essential public health functions, multisectoral factors and cross-sectoral actions. There is evidence linking people’s health and well-being to poverty reduction, education, access to clean air and water, the realization of human rights and sustainable livelihoods and environments, all of which are underpinned by good governance.^7^^,^^17^^,^^25^^,^^28^^–^^30^ The individual good and social good are mutually dependent and both are underpinned by the physical environment. Taking this interdependence into account requires a paradigm shift in health and development policies and programmes.^9^^,^^10^^,^^17^^,^^30^^,^^31^

## Implications for implementation

The implementation of a life-course approach to health involves four areas: (i) policy and investment; (ii) health services and systems; (iii) local, multisectoral and multistakeholder action; and (iv) measurement, monitoring and research.

### Policy and investment

A life-course approach requires holistic, long-term, policy and investment strategies that promote better health outcomes for individuals and greater health equity in the population – the two are interlinked.^30^ For example, since 1995 the Australian Longitudinal Study on Women’s Health has provided evidence of how gender and health inequities accumulate throughout life: women who struggled to manage financially before the age of 20 years had an increased risk of reduced physical capacity in later life and their physical decline started earlier.^15^ To counter this, the government updated its policy to ensure age-appropriate health care and address inequities across the life course.^13^^,^^15^

Long-term investment in a life-course approach can provide high returns for health and sustainable development, both by limiting ill health and the accumulation of risk throughout life and by contributing to social and economic development. For example, in 2013 it was reported that 11% of recent economic growth in low- and middle-income countries resulted from a reduction in preventable deaths.^32^ Investment in early childhood, child and adolescent health and development can yield a benefit-to-cost ratio of 10-to-1 in health, social and economic benefits and rates of mental health disorders and noncommunicable diseases in later life can be reduced.^26^^,^^27^^,^^33^^,^^34^ In 2014, an analysis indicated that a comprehensive package of family planning, quality-of-care improvements in pregnancy and childcare and the prevention and management of childhood illnesses would yield 9 United States dollars in economic and social benefits in low- and middle-income countries for every dollar spent.^35^ For older people, integrated health, social and environmental investment can help reduce health-care costs and care dependency and promote well-being.^36^ Investment in evidence-based interventions can also improve resilience and mitigate risks throughout life and across generations.^9^^,^^10^^,^^13^^,^^14^^,^^19^

Despite the evidence supporting disease prevention, in 2015 the Organisation for Economic Co-operation and Development (OECD) estimated that spending on prevention had decreased since 2009 in around half of OECD countries, whereas spending on long-term, outpatient and inpatient care continued to grow.^37^ Much more focus is required on preventing health risks and reducing their cumulative effect throughout life and across generations, and to avoid prohibitive health-care costs. Further, domestic health expenditure and development assistance for health have tended to prioritize specific diseases or life stages over others.^38^ A more holistic, long-term approach to policy and investment is required to operationalize a life-course approach to health, thereby helping to ensure UHC and realize the SDGs.

### Health services and systems

Properly implemented, a life-course approach can improve the effectiveness and reach of UHC. Planning for a life-course approach to health should coincide with national planning to achieve UHC, preferably in a people-centred manner. For example, integrating services holistically to address people’s health and well-being, rather than focusing on specific diseases or issues, can yield better health outcomes and improve efficiency.^39^ A two-year study of health sector reform in China highlighted the value of people-centred integrated care for ensuring better health for the population at all ages: quality of care was enhanced, individuals and families experienced better care and the cost was affordable for both individuals and government – all factors critical for UHC.^40^

**Fig. 2:**
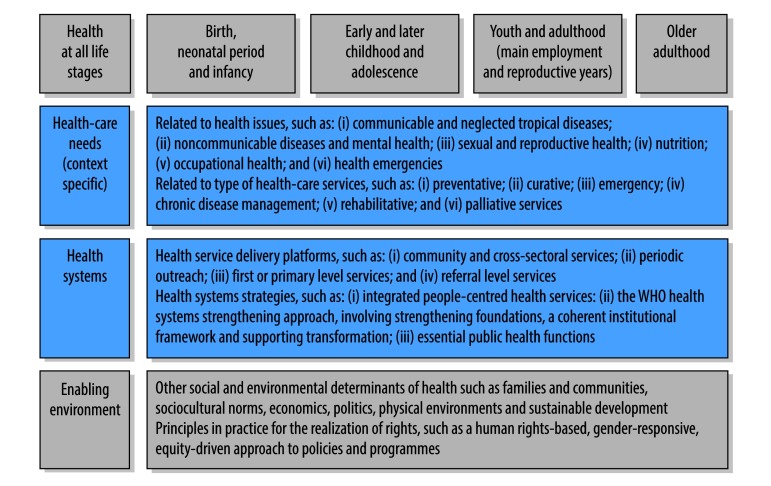
Planning universal health coverage using a life-course approach to health

Countries should themselves ascertain how best to implement UHC to ensure high-quality and accessible services and provide financial protection that matches the unique health-care needs of their populations.^19^ These needs are detailed in Fig. 2, which shows the core UHC framework, comprising health-care needs and health services and systems, and includes consideration of life stages and the enabling environment.

WHO: World Health Organization.

Notes: The blue boxes list considerations included in the core universal health coverage framework. The grey boxes list considerations intrinsic to a life-course approach to health.

Source: Adapted from *Together on the road to universal health coverage: a call to action*.^19^

Health systems strategies such as integrated people-centred health services can contribute to operationalizing the life-course approach and to implementing UHC.^41^ A strong health-care system is essential and WHO proposes an approach to improving system performance that will help it evolve and respond to emerging challenges. This approach has three elements: (i) strengthening the foundations of the health system; (ii) setting a coherent institutional framework; and (iii) supporting transformation.^42^ In addition, essential public health functions, which constitute a country’s minimum public health requirements in areas such as governance, disease control, health promotion and health research,^43^ are important for linking actions in health and other sectors. All these elements require investment in, and the development of, a dedicated health workforce.

### Local action

Global strategies and national policies have to be translated into local actions to achieve the desired outcomes. Actions across different sectors of society can have a synergistic effect in improving health, reducing poverty, improving education and gender equality and promoting socioeconomic and sustainable development.^7^^,^^25^ Cross-sectoral programmes involving health and other sectors are key. For example, the Madsen’s Institute for Tribal and Rural Advancement programme in India used a life-course approach with cross-sectoral programmes to transform the health of, and prospect of sustainable development for, people in 48 villages in Orissa.^44^ The programme, which was led by local communities along with local health services and government, started with malaria control and expanded, according to the communities’ needs, to include other health, educational, environmental and poverty-reduction goals, because these were recognized as interdependent. The result was a range of health, social and developmental advances and the infant mortality rate halved over 15 years, though it remained extremely high in neighbouring villages not covered by the programme.^44^

Policy and implementation efforts across different sectors can be coordinated by applying, for example, the Health in all Policies approach^25^ and multistakeholder policy dialogue.^45^ These techniques can help promote accountable and participatory governance and the institutionalization of cross-sectoral programmes and multistakeholder partnerships, in alignment with the SDGs. Political leadership is critical for assuring an integrative, multisectoral approach.^7^^,^^25^ Many countries have made progress towards achieving UHC and improving health and sustainable development using strategies aligned with these implementation techniques: Brazil, Chile, China, Cuba, Rwanda, Thailand and subnational regions such as Kerala in India have strengthened health systems, broadened access to health services and provided progressive financing to expand UHC.^28^^,^^46^ These countries and others made progress by also addressing inequities in, and the social and environmental determinants of, health.^7^^,^^25^^,^^28^^,^^46^

### Measurement, monitoring and research

Better understanding of the life-course approach to health, which can be obtained from both objective measures and people’s subjective experience, can guide individual and collective efforts to improve functional ability at all ages. In particular, more information is needed on how greater life expectancy in some populations affects individuals’ health and well-being in later life. Several key issues influencing measurement, monitoring and research should be considered.

#### Outcomes

New international standards incorporating a core set of indicators applicable to all life stages are needed to improve the measurement of life-course variables.^12^^,^^13^

#### Age

Chronological age may not accurately represent biological, social, psychological or functional age or an individual’s experience of ageing.^12^^,^^13^ Clearer information on age ranges for specific life stages and on how to identify critical phases and pathways would enable key services to be targeted more effectively.

#### Determinants of health

The focus should be on determinants that are modifiable or amenable to change. Determinants that shape a particular life stage or influence health throughout life should be identified and monitored.^36^

#### Change in functioning and interventions

Identify and measure both factors that can cause a decline in functioning or well-being and those that can reverse that decline (e.g. reducing air pollution) and determine how this knowledge was obtained. Identify interventions likely to produce the greatest gains at different life stages.

#### Critical pathways and complex systems

A unified measurement approach is needed to take account of the wide variety of life course variables.^10^^,^^13^^,^^14^ This should include metrics for analysing the life course, measures of people-centred services and patient-reported outcomes.

#### Equity

Increased understanding is needed of how and why some people and groups experience unequal access to services, resources and power and have restricted rights and freedoms. Data should be collected from, for example, statistical surveys, administrative records and human rights reports. Engagement with disadvantaged communities is required to assess and address inequities.^47^

#### Research studies

Substantial investment in birth cohort, longitudinal and intervention studies is needed, particularly in low- and middle-income settings.^13^^,^^14^^,^^47^ Past examples include WHO’s Study on global AGEing and adult health (SAGE) in six low- and middle-income countries^48^ and the Longitudinal Ageing Study in India (LASI).^49^ Assuring long-term investment in cohort studies across the life course can be challenging, as in the 30-year Pelotas birth cohort study in Brazil.^50^

#### Country information systems

High-quality data on health, health equity and social and environmental determinants of health across the life course are needed, along with the capacity to analyse, communicate and use the data for policies and programmes.

#### Implementation research and knowledge exchange

Research policy and new research should be aligned to support and understand the implementations of the life-course approach. The exchange of knowledge between countries should be promoted.

#### Alignment of monitoring frameworks

Table 1 provides examples of how SDGs, which have already been integrated into monitoring frameworks in many countries, can be aligned with the life-course approach. Indeed, the life-course approach is relevant to many SDGs. However, more work is required to improve the tracking of individuals’ functional ability and health trajectories, including the accumulation of resilience and risk throughout life and across generations. It will also be important to determine how countries use this information to take appropriate actions and to achieve better outcomes.

**Table 1:** Alignment of sustainable development goals with a life-course approach to health

Aspect of life-course approach to health	Sustainable development goal	Examples of alignment between the SDG and a life-course approach to health
Health and well-being	SDG 2: Zero hunger	Eliminating malnutrition and meeting the nutritional needs of children, adolescent girls and pregnant and lactating women
	SDG 3: Good health and well-being	Improving maternal and newborn health, reducing child mortality and combating HIV infection, tuberculosis, malaria, neglected tropical diseases, other communicable diseases and noncommunicable diseases and improving mental health
	SDG 4: Quality education	Ensuring early childhood development
	SDG 5: Gender equality	Ensuring universal access to sexual and reproductive health services and reproductive rights
Social and environmental determinants of health	SDG 1: No poverty	Eradicating extreme poverty, achieving coverage of basic services and appropriate new technologies and implementing nationally appropriate social protection systems and measures for all by sex, particularly for children, the unemployed, older people, people with disabilities, pregnant women, neonates, people injured at work and the poor and vulnerable
	SDG 3: Good health and well-being	Promoting universal health coverage and improving health systems by increasing both funding and the health-care workforce
	SDG 4: Quality education	Ensuring that all girls and boys complete free, equitable and good-quality secondary education
	SDG 6: Clean water and sanitation	Achieving universal and equitable access to safe and affordable drinking water and adequate sanitation and hygiene
	SDG 7: Affordable and clean energy	Ensuring universal access to affordable, reliable and modern energy services
	SDG 11: Sustainable cities and communities	Ensuring holistic, disaster risk management at all levels
	SDG 13: Climate action	Integrating climate change measures into national policies, strategies and planning
	SDG 14: Life below water	Preventing marine pollution, particularly from land-based activities, including nutrient pollution, which can get into the food chain and affect human health and the biosphere
	SDG 15: Life on land	Combating desertification, restoring degraded land and soil, promoting fair and equitable sharing of the benefits arising from the utilization of genetic resources and promoting appropriate access to such resources
	SDG 17: Partnerships for the Goals	Enhancing the global partnership for sustainable development
Principles in practice for the realization of rights	SDG 5: Gender equality	Eliminating all harmful practices, discrimination and violence against women and girls and ensuring women’s participation and equal opportunities for leadership at all levels of decision-making
	SDG 10: Reduced inequalities	Ensuring the social, economic and political inclusion of all, irrespective of age, sex, disability, race, ethnicity, origin, religion or economic or other status
	SDG 16: Peace, justice and strong institutions	Ensuring a legal identity for all (including birth registration), developing effective and accountable institutions, ensuring public access to information and protecting fundamental freedoms

HIV: human immunodeficiency virus; SDG: sustainable development goal.

#### Qualitative studies

Such studies are needed to gain an insight into the context-specific, individual, sociopolitical, cultural, economic and environmental factors that influence health and well-being throughout life, some of which may otherwise be overlooked or poorly understood.^13^

## Conclusion

A life-course approach to health can help shape UHC and wider ambitions for health and the SDGs in several ways. First, and by definition, a life-course approach is central to ensuring the health and well-being of all people at all ages (SDG 3). Second, this approach recognizes the interdependence of people, prosperity and the environment and can be employed to implement multisectoral actions for achieving shared goals. Third, it can inform country-specific planning for UHC and bring together elements of many existing national, regional and global health and development strategies. Fourth, it emphasizes rights, gender equality and equity, thereby ensuring no one is left behind – a central tenet of the 2030 agenda for sustainable development. The usefulness of the life-course approach for helping countries address critical, interdependent factors affecting health and sustainable development in a holistic manner means that it fits perfectly with efforts to achieve UHC and to realize the SDGs. However, translating this approach into actions will involve overcoming numerous constraints and will require more work by stakeholders across many sectors of society.
